# Spin-Orbit Coupling Electronic Structures of Organic-Group Functionalized Sb and Bi Topological Monolayers

**DOI:** 10.3390/nano12122041

**Published:** 2022-06-14

**Authors:** Qi Gong, Guiling Zhang

**Affiliations:** School of Material Science and Chemical Engineering, Harbin University of Science and Technology, Harbin 150040, China; gongqi.hrbust@gmail.com

**Keywords:** two-dimensional topological insulator, quantum spin Hall effect, Dirac edge state, electronic band-gap

## Abstract

Electronic band-gap is a key factor in applying two-dimensional (2D) topological insulators into room-temperature quantum spin Hall effect (QSH) spintronic devices. Employing pseudopotential plane-wave first-principles calculations, we investigate spin-orbit coupling (SOC) electronic structures of the novel 2D topological insulator series of antimony (Sb) and bismuth (Bi) monolayers (isolated double atomic layers) functionalized by organic-groups (methyl, amino and hydroxyl). Cohesive energies and phonon frequency dispersion spectra indicate that these organic-group decorated Sb and Bi monolayers possess structural stability in both energetic statics and lattice dynamics. The giant electronic band-gaps adequate for room-temperature applications are attributed to the effective SOC enhancement of group functionalization. The nontrivial topology of these novel 2D monolayer materials is verified by the Z_2_ invariant derived from wave-function parity and edge-states of their nanoribbons, which is prospective for QSH spintronic devices. The chemical functional group changes the *p*-orbital component of Fermi level electrons, leading to strong intra-layer spin-orbit coupling, opening a large band-gap of approaching 1.4 eV at Dirac-cone point and resulting in a global indirect band-gap of 0.75 eV, which, even underestimated, is adequate for room-temperature operations. Sb and Bi monolayers functionalized by organic groups are also predicted to maintain stable nontrivial topology under in-layer biaxial strain, which is suitable for epitaxy technology to realize QSH spintronic devices.

## 1. Introduction

Two-dimensional (2D) topological insulator (TI) with quantum spin Hall effect is a new class of topological quantum state matter whose electronic structure presents intrinsic time-inversion-symmetry (Z_2_ conservation) of nontrivial topology, which means its physical properties and quantum-states are not affected by material defects or impurities and will provide a potential application platform for energy-free spintronic devices [1,2]. Conductive surfaces or edge-states of Z_2_ conserved topological insulators are restrained by spin-time inversion symmetry, and the electron motions of macroscopic conduction currents rely on the electron spin states [3,4]. In 2D TI materials, electrons are not subjected to back scattering from nonmagnetic impurities, and therefore the energy loss of Joule heat will not be generated during edge-state electron transports [5]. Because the spin-orbit coupling (SOC) of carbon atoms is very weak, the Dirac-cone band-gap of graphene under SOC is only on the order of 10^−3^ meV, far from realizing 2D TI applications of room-temperature spintronic devices [6]. Graphane-like silicene, germanene, chemically modified sitene and the compound ZeTe_5_ have also been theoretically proven to be 2D TI materials. So far, only HgTe/CdTe and InAs/GaSb quantum wells have been experimentally demonstrated to be 2D TI materials with a very small bulk band-gap, which, however, can only fulfill QSH at very low temperatures and fail to be applied in room-temperature electronic devices [7,8,9,10]. Therefore, the development of a new class of 2D TI materials with adequately wide electronic band-gaps is the prerequisite for energy-free spintronic devices, which is of great significance to the collective scientific knowledge of topological quantum states.

Serrated hexagonal honeycomb 2D materials composed of antimony (Sb) or bismuth (Bi) elements have strong SOC, which are expected to become a new class of room-temperature 2D TI [11,12]. It has been proven by first-principles calculations that Sb (111) film will be transformed into 2D topological phase under planar biaxial strain, while Bi (111) film (hereinafter referred to as monolayer) is a 2D TI with a bulk band-gap of about 0.2 eV [13,14]. Although the Bi monolayer was prepared experimentally, its QSH has not been observed until now [15,16]. 2D topological materials with a large band-gap can be obtained through halination and hydrogenation, but the plasma method of experimental preparation leads to a sharp increase in lattice defects and even a serious impact on topological properties, making it difficult to achieve high-quality chemical modifications [17]. In contrast, the functionalization reactions of organic groups are relatively slow and more suitable to increase bulk band-gaps by passivating the surface conjugate bonds of 2D materials [18]. BiX (B, Al, Ga, and In) compound monolayers have been predicted by first-principles calculations to be capable of acquiring substantial improvements in their bulk band-gaps by surface hydrogenation, which even results in energy band inversions of their nanoribbons [19]. Whereas a hydrogenated surface is easily oxidized at room temperature and normal pressure due to its poor chemical stability, while the functionalized surface of organic groups has antioxidant capability and higher thermal stability for successful device applications [20,21,22]. Therefore, the chemical modifications of Sb and Bi monolayers by organic groups are preferable for realizing 2D topological materials with giant band-gaps.

In this paper, the electronic structures and topological properties of methyl, amino, and hydroxyl functionalized Sb and Bi monolayers are calculated by the first-principles pseudopotential plane-wave method. We focus on electronic band-gap, band-edge splitting, and atomic orbital components under SOC, investigating the effect of organic-group functionalizations on the electronic band-gaps and nontrivial topology of Sb and Bi monolayers according to the Z_2_ invariant and topological edge-states, exploring their potential applications in QSH electronics. The influence of in-plane biaxial strain on band-gap and topological properties are also elucidated to prove that the organic-group functionalized Sb and Bi monolayers have mechanical stability for nontrivial topology and giant band-gaps to withstand electron thermal excitations at room temperature.

## 2. Atomic Model and Calculation Schemes

Atomic structures and electronic properties of Sb and Bi monolayers functionalized with organic groups are calculated by first-principles pseudopotential plane-wave schemes, as implemented by CASTEP code of Material Studio 2020 package (Accelrys Inc., Materials Studio version 2020.08, San Diego, CA, USA). Gradient correction exchange-correlation functional PBESOL is adopted to calculate SOC-included electronic-states [23]. To reveal the SOC-produced energetic splitting of electronic-states with different spin components, the band structures without SOC are also calculated in comparison to the SOC-included band structures. The interaction of valence electrons with atomic-cores is described by norm-conserving pseudopotential, and the relativistic effect is evaluated by Koelling–Harmon treatment [24]. Electronic wave-functions are expanded by the plane-wave basis-set with a cutoff kinetic energy of 900 eV. The energy and stress are calculated under finite basis-set correction to adequately reduce the calculation error of basis-set finiteness [25]. Self-consistent field iteration in convergence of 5 × 10^−7^ eV/atom is performed by Pulay schemes of density mixing with a charge mixing amplitude of 0.5, in which electron density is calculated on a high-resolution FFT grid of 40 × 40 × 216 [26,27]. The *k*-point sampling of Brillouin zone integration is carried out on Monkhorst-Pack 5 × 5 × 1 grid [28]. Atomic-structure relaxation is achieved by geometrical optimization of energy functional minimization using LBFGS algorithm to obtain energy convergence of 5.0 × 10^−^^6^ eV/atom so that atomic interaction force and internal stress are less than 0.01 eV/Å and 0.02 GPa, respectively [29]. Phonon structure (phonon frequency dispersion spectrum) is calculated by the linear response method [30], in which the convergence standard of mechanical constant is set as 1.0 × 10^−5^ eV/Å^2^, and the non-analytical correction is applied to calculate the frequency-splitting of longitudinal-transverse optical phonons at G point.

## 3. Results and Discussion

### 3.1. Atomic Structure

**Figure 1:**
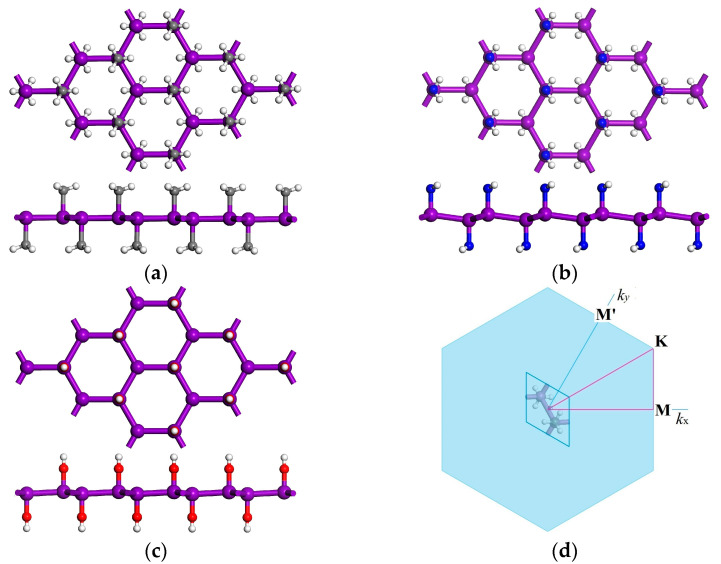
(**a**–**c**) Schematics of SbXH_n_ and BiXH_n_ monolayers decorated with methyl, amino, and hydroxyl groups, respectively, in which violet, gray, blue, red, and white balls symbolize the bonding atoms of Sb/Bi, carbon, nitrogen, oxygen, and hydrogen, respectively; (**d**) high symmetry points in the dispersion paths of electronic energy and phonon frequency in Brillouin zone.

Atomic structures of Sb and Bi monolayers with chemical decorations of methyl, amino, and hydroxyl functional groups (SbXH_n_ and BiXH_n_: XH_n_ = CH_3_, NH_2_, OH) are shown in Figure 1 where the high-symmetric dispersion paths in Brillouin zone are also exhibited. The *p*-orbitals of Sb and Bi atoms are in sp^3^ hybridization with methyl, amino, or hydroxyl groups bonded to the unsaturated *p*_z_-orbitals of Sb or Bi atoms on the surfaces of monolayers. The 2D crystal structures of SbXH_n_ and BiXH_n_ incorporate two Sb or Bi bonding atoms and two identical chemical groups into one primitive unit-cell of trigonal point group D_3d_ or monoclinic C_2h_, with a space symmetry group of P-3M1 or C2/M.

Space symmetry groups, lattice constants, cohesive energies, bond lengths, warping thicknesses of Sb or Bi atomic-layers, and total monolayer thicknesses of SbXH_n_ and BiXH_n_ in geometry-optimized structures are listed in Table 1. Cohesive energies are calculated by formula *E*_coh_ = 2(*E*_m_ + *E*_group_) − *E*_unit_, where *E*_m_, *E*_group_ and *E*_unit_ denote total energies of the isolated Sb or Bi atoms, the chemical groups, and the primitive unit-cells of SbXH_n_ or BiXH_n_, respectively. The cohesive energies of SbXH_n_ and BiXH_n_ approach 13~16 eV/unitcell, which is overall higher than that of TMD and III-VI compound monolayers [31,32,33], implying their energetic stability in statics.

**Figure 2:**
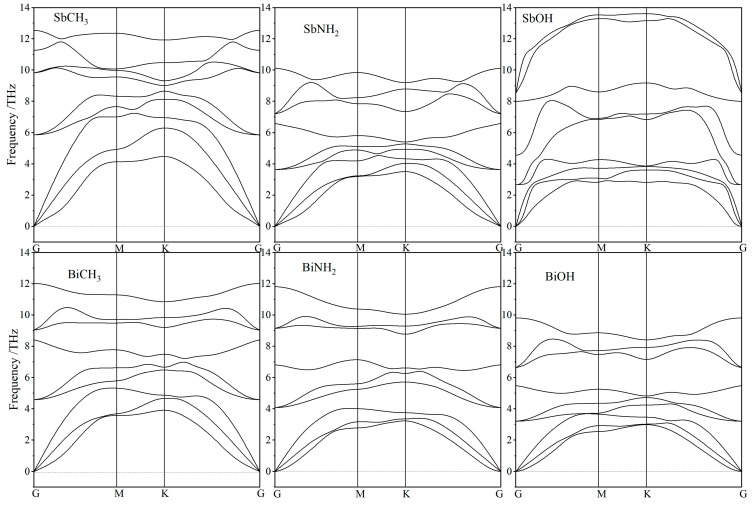
Phonon frequency dispersion spectra of SbXH_n_ and BiXH_n_ in atom-relaxed structures.

Kinetic stabilities of SbXH_n_ and BiXH_n_ are evaluated by the phonon dispersion spectra of their relaxed atomic structures (after geometrical optimization), as shown in Figure 2. Their phonon dispersion curves are comprised of 9 branches, including 3 acoustic branches and 6 optical branches. Three kinds of acoustic phonon modes are longitudinal waves, in-plane transverse waves, and off-plane transverse waves (from high frequency to low). The six optical branches contain two sets of optical waves, each incorporating a non-degenerate off-plane transverse mode and a pair of G-point degenerate longitudinal and in-plane transverse modes. Neither SbXH_n_ nor BiXH_n_ represent any virtual frequencies, i.e., all the intrinsic frequencies of phonon modes are positive, demonstrating that they are kinetically stable two-dimensional structures.

### 3.2. Band Structure and Topological Property

**Figure 3:**
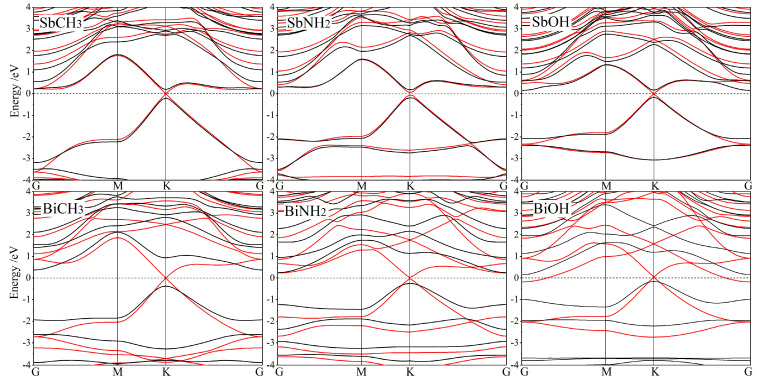
Band structures of SbXH_n_ and BiXH_n_ with SOC (black curves) and without SOC (red curves), in reference to Fermi level (horizontal dash lines).

In the absence of SOC, the methyl or hydroxyl modified Sb and Bi monolayers represent a metallic band structure, with the lowest conduction and highest valence bands crossing on Fermi level at K point, as shown by red curves in Figure 3. For SbXH_n_ and BiXH_n_, the linear energy-***k*** dispersion (constant first derivative) at band-edges characterizes the typical Dirac-cone point, similar to graphene and halogenated Bi monolayers, but the orbital compositions of electronic-states at Dirac-cone point are significantly discrepant from that of pure Bi monolayer. Moreover, without SOC, Dirac-cone points of SbNH_2_ and BiNH_2_ show band inversions, forming band-gaps of 0.112 eV and 0.050 eV, respectively. There are two (even) bonded hydrogen atoms on one amine group, which leads to C_2h_ point-symmetry of SbNH_2_ and BiNH_2_, indicating that a pair of degenerate electronic-states at high symmetry points in band structures will split into two energy levels; that is, two crossing energy dispersion curves at high symmetry points will split into two energy bands of upper and lower concave. Therefore, in the absence of SOC, degenerate states of Dirac-cone point at K point undergo energy level splitting to open a non-degenerate band-gap, in which the resulted band-edge electronic-states have mixed components of conduction and valence bands, accounting for energy band inversions.

SOC determines band structures near Fermi levels of SbXH_n_ and BiXH_n_, which splits the degenerate energy levels of conduction and valence bands at Dirac-cone point to form significant band-gaps. Therefore, by including SOC, the SbXH_n_ and BiXH_n_ represent as the direct and indirect band-gap semiconductors, respectively, as shown by black curves in Figure 3. This SOC-introduced feature is similar to 2D TI monolayers of silene, germanene, and ZeTe_5_, while SbXH_n_ and BiXH_n_ give rise to giant bulk band-gaps (the highest value approaching 0.745 eV as shown in Table 2) remarkably larger than ~0.2 eV of pure Bi monolayer, which means a more preferable stability for realizing exotic quantum effects such as QSH at room temperature. Since the GGA functional always underestimates DFT band-gaps generally by about 40% [34], the real band-gaps of SbXH_n_ and BiXH_n_ will somehow be higher than the calculated values reported in this paper. Even underestimated, the predicted giant bulk band-gaps (>0.3 eV) can still adequately impede electron thermal-excitation at room temperature, indicating the feasible realizations of topological electronic devices by SbXH_n_ and BiXH_n_.

The obvious deformation of Dirac-cone under SOC indicates the presence of a topologically nontrivial phase where the giant band-gaps also originates from SOC, implying that SbXH_n_ and BiXH_n_ are likely nontrivial topological insulators, but still requiring TI characteristics of band structure represented by Z_2_ invariant and edge-states. Eigenvalues (topological index) *ν* = 1 and *ν* = 0 of Z_2_ topological invariant characterize the nontrivial topological phase (topological insulator) and the trivial topological phase (normal insulator), respectively. Accordingly, the topological properties of SbXH_n_ and BiXH_n_ are analyzed to determine whether they are topologically nontrivial. Since the atomic structures of SbXH_n_ and BiXH_n_ possess space inversion symmetry, their Z_2_ topological invariants can be calculated directly from the parities of Bloch wave-functions of the occupied electronic-states at all the time-reversal-invariant points (TRIP). Electronic structures of SbXH_n_ and BiXH_n_ have four TRIP, one at G point and three at M point, so the topological index *P* can be calculated as follows:

(1) $$P(K_{i})=\prod_{m=1}^{N}\delta_{2m}^{i},\;{\left(-1\right)}^{\nu }=\prod_{i=1}^{4}P(K_{i})=P(G)P{\left(M\right)}^{3}$$

where *P* denotes the parity product of Bloch wave-functions on TRIP, *δ* = ±1 indicates parity, and *N* is the number of valence bands. The 24 valence electrons in primitive unit-cell of SbXH_n_ or BiXH_n_ constitute 12 spin-degenerate levels at TRIP, and the parities of spin-degenerate electron eigen-functions of SbXH_n_ and BiXH_n_ on TRIP are identical. As shown in Table 3, the values of *P* at G and M points are −1 and +1, respectively, which leads to a nontrivial topological invariant of Z_2_ = 1, demonstrating that SbXH_n_ and BiXH_n_ are nontrivial topological insulators.

**Figure 4:**
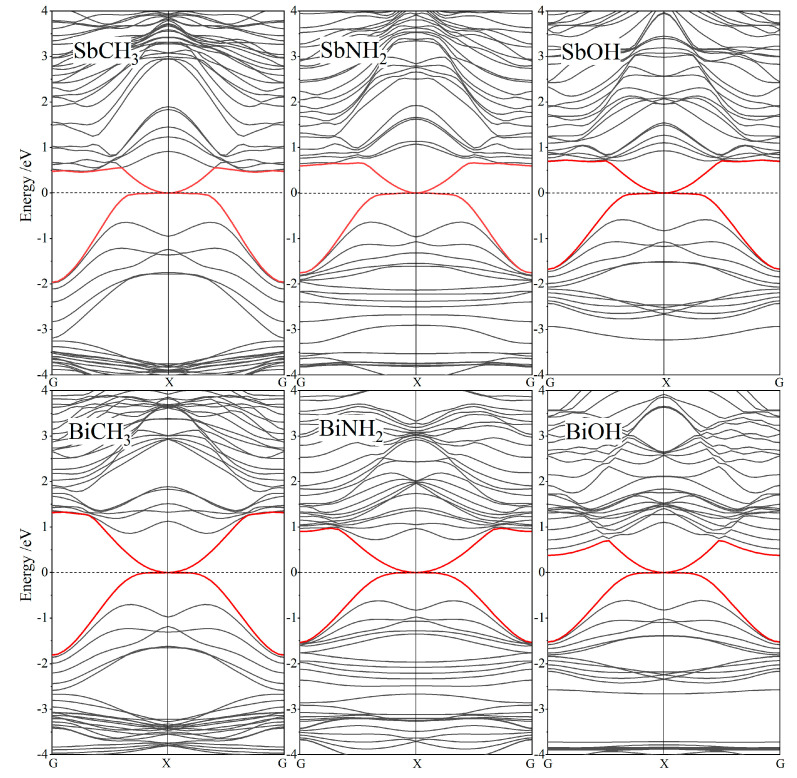
The SOC-incorporated band structures of SbXH_n_ (**top panels**) and BiXH_n_ (**bottom panels**) zigzag nanoribbons in reference to Fermi level as energy zero (horizontal dash lines).

Nontrivial topological properties of SbXH_n_ and BiXH_n_ should, meanwhile, be manifested by the odd number of conductive channels from zero band-gap edge-states, where Dirac-cone points connecting conduction and valence bands at Fermi level should appear in band structures of their nanoribbons. The zigzag nanoribbons of SbXH_n_ and BiXH_n_ are modeled with a mirror-symmetry around ribbon center axis and the edge unsaturated bonds being hydrogenated. The nanoribbon width is specified approaching ~30 Å to prohibit overlapping of electronic wave-functions between two nanoribbon edges. After geometrical optimizations, the band structures of SbXH_n_ and BiXH_n_ nanoribbons are calculated, with the results being shown in Figure 4.

Energy dispersion curves of nanoribbon edge-states intersect through the Dirac-cone point on Fermi level at the boundary X point of one-dimensional Brillouin zone. The odd number of edge-state conductive channels derived from the nontrivial topology will also present edge-state Dirac-cones in band structures of SbXH_n_ and BiXH_n_ arm-chair nanoribbons. To this end, these Dirac-cones of edge states demonstrate that SbXH_n_ and BiXH_n_ are nontrivial topological insulators which are supported by their giant-band-gaps to realize QSH effect at room temperature.

Although the SOC strengths of organic groups used for chemical modifications on Sb and Bi monolayers are almost negligible, the resulted band-gaps caused by SOC are significantly increased. For example, the bulk band-gap of pure Bi monolayer is only 0.2 eV, while the SOC band-gap is increased to 0.745 eV by methyl functionalization. Both the pure Sb or Bi monolayer and SbXH_n_ or BiXH_n_ (XH_n_≠NH_2_) possess *p*-orbital components of electronic-states near Fermi level that could be distinguished into *p*_z_ and *p*_x/y_ where the SOC intensity of *p*_x/y_-orbitals is much higher than that of *p*_z_-orbitals. The Fermi-level electronic-states of Sb or Bi monolayer are mainly derived from *p*_z_-orbitals of Sb or Bi atoms (similar to graphene and silene), while Fermi-level orbital components of SbXH_n_ or BiXH_n_ originates dominantly from *p*_x/y_-orbitals of Sb or Bi atoms. Chemical decorations of organic groups (functionalization) eliminate the delocalized conjugate π bonds of Sb or Bi *p*_z_-orbitals near Fermi level and produce the localized *σ* bonds below Fermi level by the functionalized groups. Therefore, the Fermi-level electronic-states of SbXH_n_ and BiXH_n_ are dominated by *p*_x/y_-orbital components, accounting for the evident enhancement of SOC to acquire giant bulk band-gaps.

**Figure 5:**
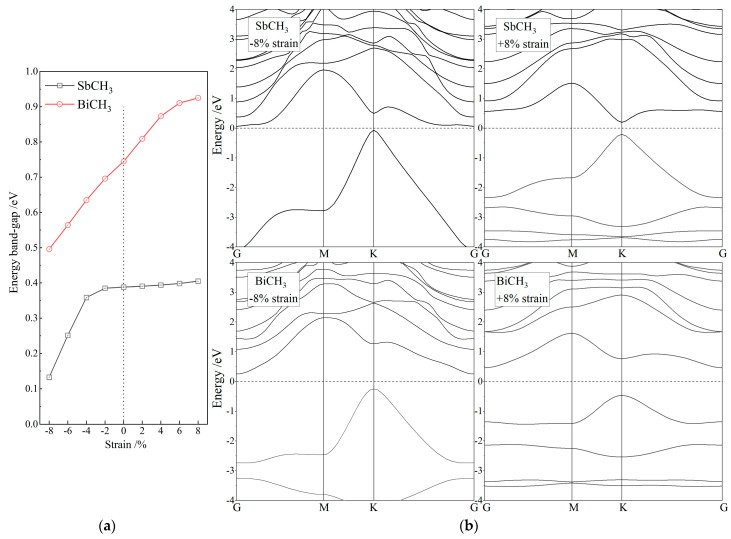
(**a**) Global bulk band-gaps in dependence on bi-axial strains of −8~8% and (**b**) band structures under ±8% bi-axial strains for SbCH_3_ and BiCH_3_, which are calculated with SOC included.

Chemical modifications of organic groups result in a significant increase in lattice constants of Sb and Bi monolayers, as shown in Table 1. The band-gap improvement under planar biaxial tension is due to that SOC strength increases with the bonding distance between Sb or Bi atoms. Thus, the increment of lattice constant or bonding distance of strong SOC atoms by chemical functionalizations of organic groups is another important attribute to the large band-gaps of SbXH_n_ and BiXH_n_. Compared to Sb and Bi monolayers, the lattice constants of SbXH_n_ and BiXH_n_ exceed by 30%, while their giant band-gaps and nontrivial topological properties do not change significantly with a strain below ±8%. SbXH_n_ and BiXH_n_ retain their nontrivial topology under biaxial strains approaching ±8%, and their band-gaps decrease by less than 10% under biaxial strains below ±4%, as shown in Figure 5. Tensile and compressive strains cause, respectively, the rise and fall of a conduction band minimum at G point with respect to Fermi level. As a result, the tensile strain evidently bears on the band-gaps of BiXH_n_ with indirect band structures, but not on the direct band-gaps of SbXH_n_. In contrast, after the compressive strain increases to −4%, the direct band-gaps of SbXH_n_ alternate to indirect band-gaps, and thus their global band-gap varies more intensively with compressive strain than that of BiXH_n_. The giant band-gap and nontrivial topology of SbXH_n_ and BiXH_n_ are quite stable in regards to mechanical deformations, indicating they are robust in broad mechanical and thermal conditions for experimental tests and spintronic device applications.

## 4. Conclusions

Electronic structures and topological properties of Sb and Bi monolayers functionalized with organic groups (SbXH_n_ and BiXH_n_, X = C, N, O) are studied by first-principles calculations in the consideration of SOC. Cohesive energies and phonon dispersion spectra indicate that these monolayered systems are innate of both statically and kinetically stable in atomic structures. Topologically nontrivial band-structures and nanoribbon edge-states prove that SbXH_n_ and BiXH_n_ are intrinsic two-dimensional topological insulators with the largest bulk band-gap approaching 0.75 eV. Due to the much higher SOC strength of *p*_x/y_ orbitals of Sb and Bi atoms than that of *p*_z_-orbitals, the organic group modifications of Sb and Bi monolayers make the dominant *p*-orbital component at Fermi level alter from *p*_z_ to *p*_x/y_, which results in a significant increase in their electronic band-gaps. Z_2_ topological invariant and Dirac-cone edge-states consistently demonstrate the nontrivial topology of SbXH_n_ and BiXH_n_. In-plane biaxial strains being increased to ±8% leads to no significant change in bulk band-gap, under which the topologically nontrivial properties of SbXH_n_ and BiXH_n_ still remain. Stable two-dimensional topological insulators with giant electronic band-gaps are predicted to be achieved by functionalizing Sb and Bi monolayers with organic groups, which provides a theoretical basis and strategy for developing novel quantum topological materials. The present study suggests SbXH_n_ and BiXH_n_ are valuable of great scientific interest and prospective of potential applications in topological electronic devices.

## Figures and Tables

**Table 1:** Space symmetry, lattice constants *a*, chemical bonding lengths (*d*_MM_ and *d*_MX_: M = Sb, Bi; X = C, N, O), thicknesses of the internal Sb/Bi layer and the entire monolayer (vertical distances between two Sb/Bi atomic-planes *h*_m_ and between the outermost two hydrogen atomic-planes on two surface sides *h*_t_), and cohesive energy (*E*_coh_) of SbXH_n_ and BiXH_n_ compared to Sb and Bi monolayers.

Monolayers	Space Symmetry	*a*/Å	*d*_MM_/Å	*d*_MX_/Å	*h*_m_/Å	*h*_t_/Å	*E*_coh_/(eV·unitcell^−1^)
Sb	P6/MMM	4.744	2.739	–	–	–	8.728
SbCH_3_	P-3M1	5.020	2.898	2.099	0.058	5.005	14.367
SbNH_2_	C2/M	4.965	2.894	1.955	0.017	4.984	15.293
SbOH	P-3M1	5.016	2.896	1.769	0.044	5.485	16.232
Bi	P6/MMM	4.960	2.864	–	–	–	7.998
BiCH_3_	P-3M1	5.309	3.066	2.225	0.067	5.244	12.943
BiNH_2_	C2/M	5.455	3.184	2.134	0.479	5.547	13.520
BiOH	P-3M1	5.289	3.059	1.917	0.173	5.928	14.501

**Table 2:** The SOC-introduced Dirac-cone splitting-gaps at K point *E*_D_(K) and the resulted indirect band-gaps from K point to G point *E*_g_(G-K) of SbXH_n_ and BiXH_n_.

Monolayers	*E*_D_ (K)	*E*_g_ (G-K)	Monolayers	*E*_D_ K)	*E*_g_ (G-K)
SbCH_3_	0.388	-	BiCH_3_	1.312	0.745
SbNH_2_	0.370	-	BiNH_2_	1.396	0.498
SbOH	0.311	-	BiOH	1.348	0.309

**Table 3:** Parities *δ* and their product *P* of spin-degenerate states at TRIP for SbXH_n_ and BiXH_n_.

TRIP	Parity *δ*												*P*
G	+1	+1	+1	−1	−1	−1	+1	+1	−1	+1	−1	+1	−1
3 M	+1	−1	−1	+1	+1	−1	−1	+1	+1	−1	+1	−1	+1

## Data Availability

Theoretical methods and results are available from all the authors.
